# Speciation and Gene Flow between Snails of Opposite Chirality

**DOI:** 10.1371/journal.pbio.0030282

**Published:** 2005-08-09

**Authors:** Angus Davison, Satoshi Chiba, Nicholas H Barton, Bryan Clarke

**Affiliations:** **1**Graduate School of Life Sciences, Tohoku University, Aramaki-Aza-Aoba, Aoba-ku, Japan,; **2**Institute of Evolutionary Biology, School of Biological Sciences, University of Edinburgh, Edinburgh, United Kingdom,; **3**Institute of Genetics, School of Biology, University of Nottingham, Nottingham, United Kingdom,; **4**Department of Genetics, University of Cambridge, Cambridge, United Kingdom; University of SheffieldUnited Kingdom

## Abstract

Left-right asymmetry in snails is intriguing because individuals of opposite chirality are either unable to mate or can only mate with difficulty, so could be reproductively isolated from each other. We have therefore investigated chiral evolution in the Japanese land snail genus *Euhadra* to understand whether changes in chirality have promoted speciation. In particular, we aimed to understand the effect of the maternal inheritance of chirality on reproductive isolation and gene flow. We found that the mitochondrial DNA phylogeny of *Euhadra* is consistent with a single, relatively ancient evolution of sinistral species and suggests either recent “single-gene speciation” or gene flow between chiral morphs that are unable to mate. To clarify the conditions under which new chiral morphs might evolve and whether single-gene speciation can occur, we developed a mathematical model that is relevant to any maternal-effect gene. The model shows that reproductive character displacement can promote the evolution of new chiral morphs, tending to counteract the positive frequency-dependent selection that would otherwise drive the more common chiral morph to fixation. This therefore suggests a general mechanism as to how chiral variation arises in snails. In populations that contain both chiral morphs, two different situations are then possible. In the first, gene flow is substantial between morphs even without interchiral mating, because of the maternal inheritance of chirality. In the second, reproductive isolation is possible but unstable, and will also lead to gene flow if intrachiral matings occasionally produce offspring with the opposite chirality. Together, the results imply that speciation by chiral reversal is only meaningful in the context of a complex biogeographical process, and so must usually involve other factors. In order to understand the roles of reproductive character displacement and gene flow in the chiral evolution of *Euhadra,* it will be necessary to investigate populations in which both chiral morphs coexist.

## Abstract 2

Is single gene speciation possible in snails? Although feasible, the authors reveal that reproductive isolation between chiral morphs is possible, but unstable, and will lead almost inevitably to gene flow.

## Introduction

Left-right asymmetry is an integral part of the establishment of a body plan that may ultimately be traced back to a much deeper molecular asymmetry [1,2]. Although substantial progress has been made recently towards understanding the establishment of asymmetry in several organisms [3–5], almost nothing is known about the first stages in any embryo. Snails may be crucial tools for studying left-right asymmetry, because their chirality is determined at a very early cleavage, and because several species have morphological variation, so that the gene(s) involved can be mapped [6]. It is intriguing that molluscan asymmetry is determined by the effects on the developing embryo of a maternal “chirality” gene (or series of closely linked loci). The genotype of the mother determines the phenotype of the offspring [7–11].

Some recent work has focussed on the issue of why snails are almost always invariant in chirality. One approach has been to study the exceptions, species that are chirally dimorphic, but even in these most populations are fixed for a particular type [11–14] (with the exception of *Amphidromus* [15]). This is because positive frequency-dependent selection tends to drive the chiral majority to fixation; rarer snails of opposite chirality are less likely to find a mate and successfully copulate [13]. In the best-studied case, the Polynesian tree snail *Partula suturalis,* dimorphic populations occurred in narrow clines between areas of dextral or sinistral snails [12,13]. In “no choice” experiments, interchiral matings occurred only 20% as frequently as intrachiral matings, and fewer young were produced by the mixed pairs [13]. Since dextral populations of *P. suturalis* tended to coincide geographically with closely related sinistral species, it was suggested that dextral *P. suturalis* became established because of reproductive character displacement: Dextral individuals were favoured even when rare, because they were less likely to waste time (or gametes) mating with sinistral species [12,13,16].

More general aspects of shell morphology in land snails have been of interest since the observation by Cain that members of widely separate and taxonomically distinct land snail faunas tend to be either high- or low-spired, with relatively few globular forms in between [17]. Following the suggestion of Gittenberger that there is an association between shell shape and the degree of variation in asymmetry [18], Asami et al. went on to report that reciprocal mating between dimorphic low-spired snails is not usually possible, because the genitalia of a sinistral individual cannot engage with those of a dextral snail [14]. In contrast, high-spired dimorphic snail species such as *Partula* are able to mate, albeit with some behavioural adjustments [14,19,20].

Interchiral mating in snails, in conjunction with the maternal inheritance of the gene, has attracted attention because it has been thought capable of producing “single-gene speciation” in sympatry ([18,21–24], but see [25]). Simulations have been used to establish whether chirally reversed snails can reach a sufficiently high density to overcome the effects of positive frequency-dependent selection. Johnson et al. considered that sympatric speciation due to coil reversal was unlikely in *Partula*, because new variant snails would have difficulty finding partners of the same chiral morph, and because the maternal inheritance allowed free gene flow between the morphs [25]. However, simulations by Orr [23], van Batenburg and Gittenberger [26], and Stone and Björklund [27] suggested that in some other circumstances single-gene speciation could occur. It is interesting that the establishment of a new chiral morph in snails has a parallel in Müllerian mimetic species, where new models can only become established when they reach a threshold density [28,29]. The positive frequency-dependent selection that creates sharp clines in the frequencies of *P. suturalis* chiral morphs could similarly maintain the narrowness of hybrid zones between geographic races of *Heliconius* butterflies [28].

**Figure 1:**
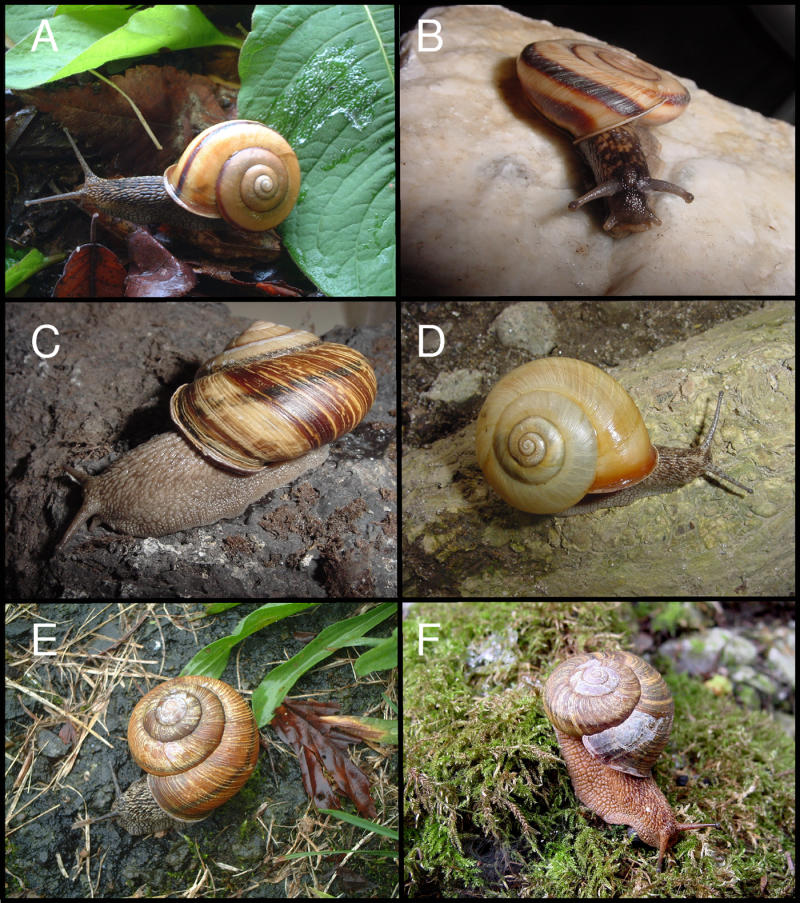
Sinistral Species (A) *E. quaesita* from Sendai and (B) *E. murayamai* from Myojo-san (in cave); dextral (C) *E.* *senck.* *senckenbergiana* from Imajyo, (D) *E. senck.* *amoriensis* from Tamayama, (E) *E. senck.* *amoriensis* from Iide-san, and (F) *E. senck. ibukicola* from Mt. Fujiwara. [Photos: AD and SC]

**Figure 2:**
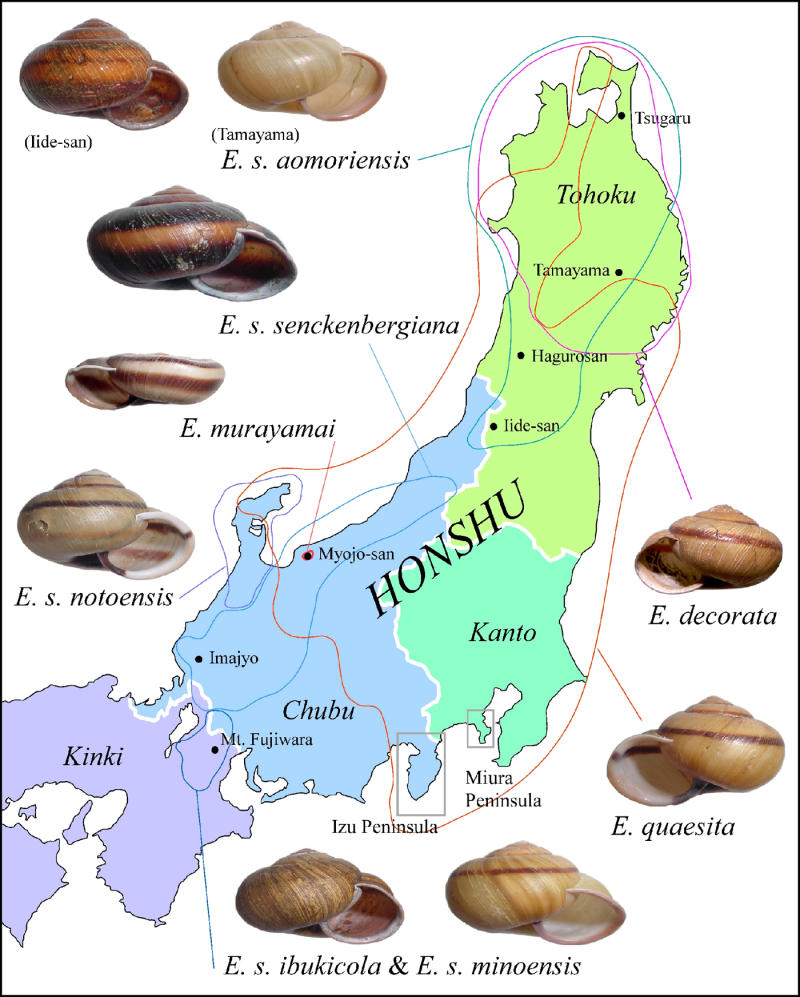
Distributions of Four *Euhadra* Species on the Main Japanese Island of Honshu *E. senck.* *aomoriensis* specimens that were used for DNA analysis were collected from Tamayama, Tsugaru, and Iide-san. *E. murayamai* is confined to a single mountain (Myojo-san). The remaining marked sites refer to sites of snails in Figure 1, as well as to others that are specifically referred to in the main text. Sinistral species are shown in red and dextral species in blue.

We chose to investigate the Japanese land snail genus *Euhadra* (Bradybaenidae) (Figure 1), precisely because it is the best candidate for single-gene speciation. Five of the 22 *Euhadra* species are sinistral (Figures 1 and 2), a situation that is particularly unusual because most other low-spired snail genera are invariant for their chirality. If single-gene speciation can ever occur, then it is most likely to occur in *Euhadra,* because interchiral mating is either difficult or impossible [14]. We were interested to know how many times sinistral *Euhadra* have evolved, and whether chiral variation has promoted speciation. We tested these ideas using a phylogeny of mitochondrial DNA gene fragments from 19 of the species, including several representatives from each of the sinistral taxa (Tables 1 and S1). As it was necessary to understand the conditions under which genes might flow between different chiral morphs (i.e., whether reproductive isolation and single-gene speciation is theoretically possible), and to make predictions of the genealogical patterns that we expect to see, we have developed a mathematical model of the population genetics of chirality (Protocol S1).

**Table 1:**
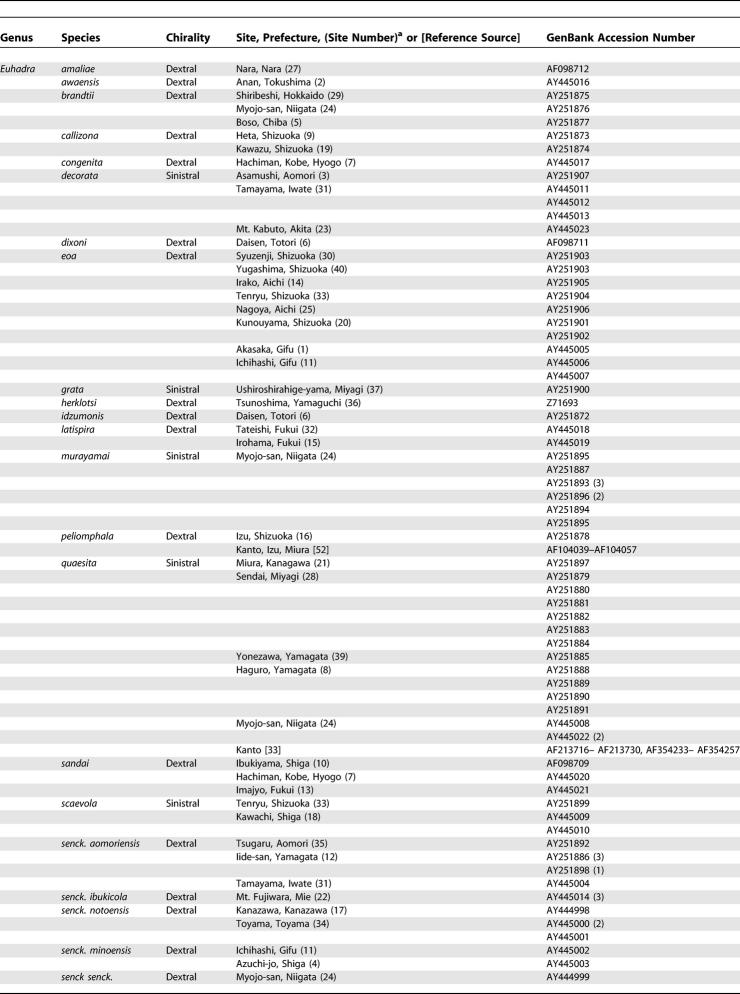
Species and Collection Localities of Samples Used for DNA Analysis

**Table 1:**
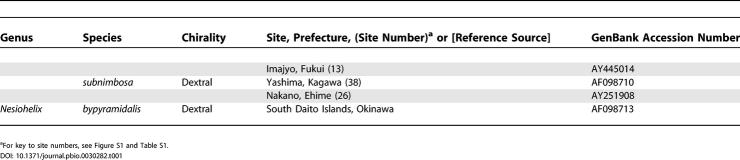
Continued

## Results

### Phylogenetic Analysis of Gene Sequences

As expected from earlier studies [30–32], mitochondrial variation was extreme both within and between species. Only the 16S rRNA fragment had a level of variation suitable for resolving both the deeper relationships and those within species (881 bp when aligned, of which 639 bp were used for the phylogeny; for GenBank references, see Table 1). The variation within 12S rRNA was confined to several hypervariable regions. Cytochrome oxidase *c* synonymous nucleotide positions were saturated and its amino acids barely variable. There was also not enough variation within the nuclear sequence of ITS2. These latter three gene fragments were therefore not used further (GenBank AY445024–AY445027, AY251858–AY251871, and AY251820–AY251837).

**Figure 3:**
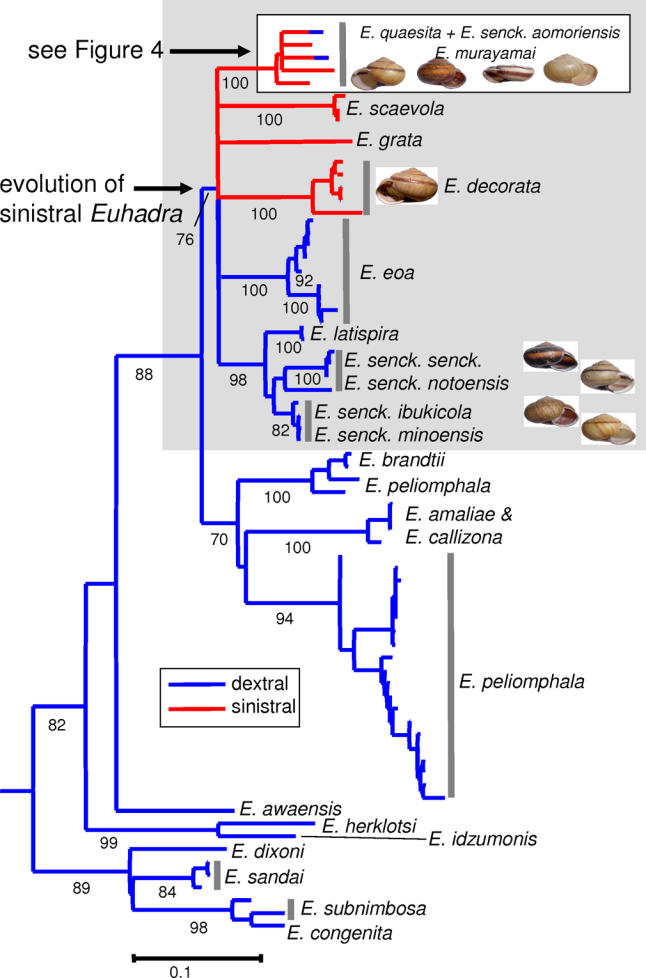
Neighbour-Joining Phylogeny 16S rRNA rate-corrected neighbour-joining phylogeny, rooted using *Nesiohelix bipyramidalis,* showing the relationship between mitochondrial DNA lineages from dextral and sinistral *Euhadra*. The most parsimonious explanation is a single, relatively ancient evolution of sinistral snails. Lineages within the box are shown in detail in Figure 4. Bootstrap support of more than 70% is shown below the node. Shape parameter = 0.30.

The sinistral species *Euhadra quaesita* (Figure 1A), *E. decorata,* *E. grata,* *E. scaevola,* and *E. murayamai* (Figure 1B) were confined to a single clade in the 16S rRNA neighbour-joining phylogeny (Figure 3), which also contained the dextral species *E. eoa,* *E. senckenbergiana* (*E. senck.;* see Figure 1C–1F), and *E. latispira*. Bootstrap support for the branch that defined this group is 76%, so the simplest explanation is that sinistral *Euhadra* evolved once only. The maximum likelihood phylogeny, using representative sequences from each clade, had the same overall structure, and the same as that discovered by Ueshima and Asami using a different mitochondrial gene fragment [24].

**Figure 4:**
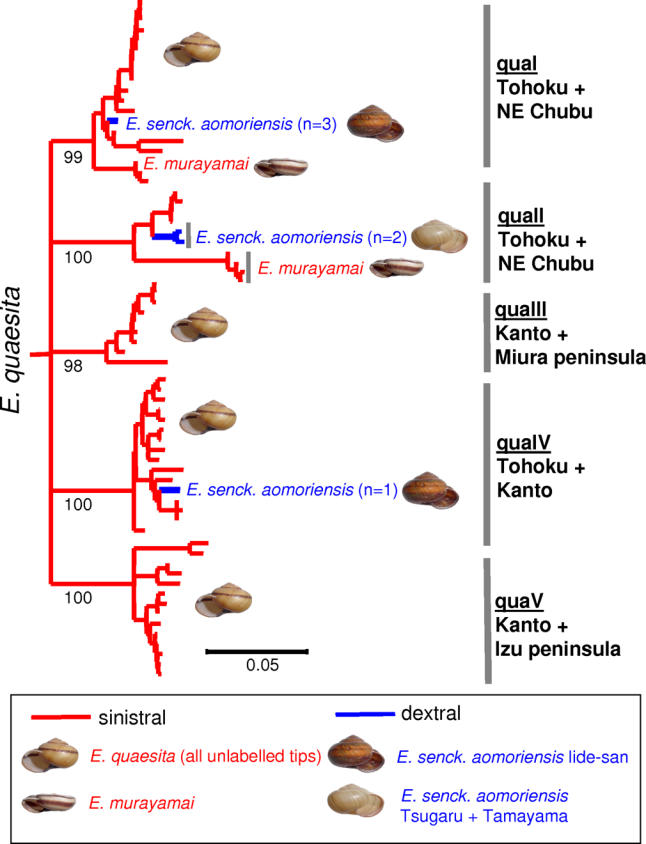
Neighbour-Joining Phylogeny Subtree 16S rRNA rate-corrected neighbour-joining phylogeny (subtree from Figure 3) showing the relationship between *E. quaesita,* *E. senck*. *aomoriensis,* and *E. murayamai* mitochondrial lineages. All unlabelled tips are *E. quaesita*. Both *E. senck*. *aomoriensis* and *E. murayamai* are polyphyletic. The polyphyly of *E. senck*. *aomoriensis* can be explained by either single-gene speciation or mitochondrial introgression. Bootstrap support shown for important nodes only.

The relationships within the clade that contains the sinistral species were surprisingly complicated (Figures 3 and 4). Six lineages were found: individual *E. decorata,* *E. eoa,* *E. grata,* and *E. scaevola* lineages, plus two multi-species lineages, one comprising two species, *E. senck.* and *E. latispira,* and the other three species, *E. quaesita,* *E. senck.,* and *E. murayamai*. The latter lineage group divides into five subgroups (Figure 4), two that have not been discovered before (QUAI and II), and three already reported (QUAIII, IV, and V) [33]. The geographic distributions of these subgroups are principally Tohoku and North East Chubu (QUAI and II), Kanto and Miura peninsula (QUAIII), Kanto and Tohoku (QUAIV), and Kanto and Izu peninsula (QUAV). All the major lineages within the clade that contains the sinistral species were strongly supported by bootstrapping, but their relationship to each other was not resolved (Figures 3 and 4).

Mitochondrial lineages of sinistral *E. murayamai* from Myojo-san, as well as dextral *E. senck.* *aomoriensis* from Iide-san, Tamayama, and Tsugaru were within the *E. quaesita,* *E. senck.,* and *E. murayamai* mitochondrial clade (Figure 4; DNA sample sites and distributions are shown in Figure 2, with further details in Figure S1 and Table S1). *E. murayamai* is found on a single mountain, yet its mitochondrial haplotypes were found in QUAI and II, nested with haplotypes from *E. quaesita*. Individual haplotypes of dextral *E. senck.* *aomoriensis* were found in QUAI, II, and IV (*n* = 6), three clades of which are predominantly found in Tohoku, but also North East Chubu and Kanto. The other subspecies of *E. senck.* grouped with *E. latispira*. Within the QUAII lineages (Figure 4), snails were therefore either sinistral *E. murayamai* from Myojo-san (North East Chubu), sinistral *E. quaesita* from Haguro-san (central Tohoku), or dextral *E. senck.* *aomoriensis* from Tsugaru and Tamayama (northern Tohoku).

### Analysis of Morphological Characters

**Figure 5:**
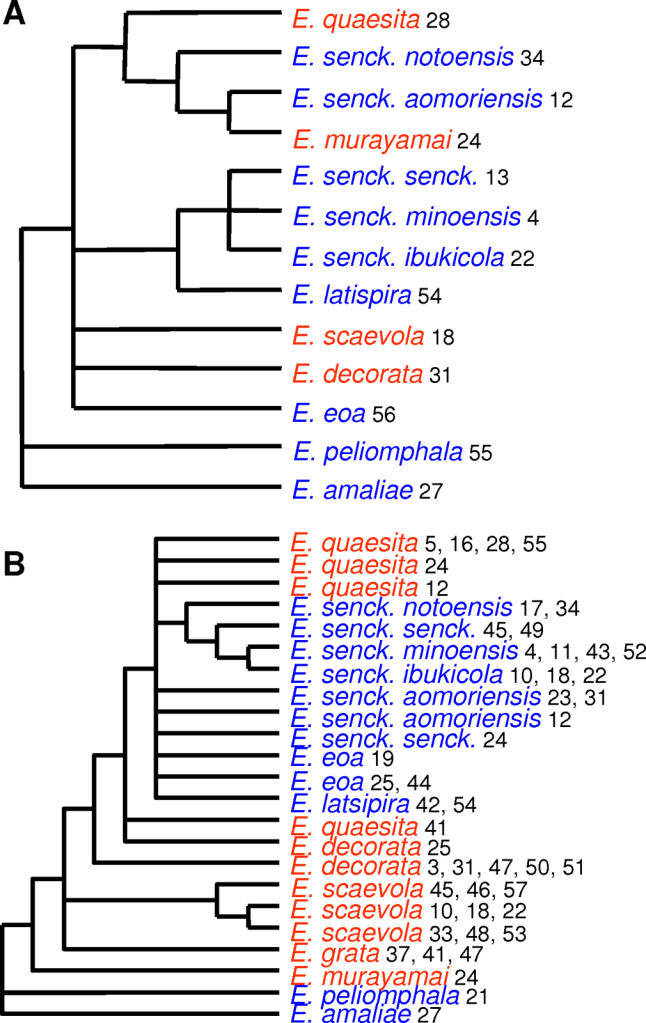
Maximum Parsimony Tree of Two Characters The tree is based on (A) genital characters and (B) shell characters. The number after each species is the sample location (see also Tables S1–S3). Sinistral species are shown in red and dextral species in blue.

The morphological analysis showed that both *E. senck. aomoriensis* and *E. senck. notoensis* have genitalia similar to those in *E. quaesita* and *E. murayamai* (Table S2). Specifically, ten of 18 *E. senck. aomoriensis* genital characters were identical to those of *E. quaesita* and different from those of *E. senck. senckenbergiana,* whereas only one of 18 was similar to *E. senck. senckenbergiana* and different from *E. quaesita* (Table S2). The morphology of the genitalia in the other *E. senck.* subspecies *(E. senck. senckenbergiana,* *E. senck. minoensis,* and *E. senck. ibukicola)* was similar to that of *E. latispira*. *E. senck. aomoriensis,* *E. senck. notoensis, E. quaesita,* and *E. murayamai* grouped together in the genital character maximum parsimony tree, distinct from the other *E. senck.* subspecies as well as from *E. latispira* (Figure 5A). The analysis of shell characters showed that there is considerable variation between populations (Figure 5B; Table S3). Consequently, different populations of the same species group closely with other species in the parsimony analysis of shell characters (Figure 5B).

In summary, *E. senck. aomoriensis* and *E. quaesita* specimens grouped together in the mitochondrial DNA phylogeny and have similar genitalia. In contrast, while *E. senck. notoensis* samples have similar genitalia to *E. senck. aomoriensis* and *E quaesita,* their mitochondrial DNA sequences grouped with the other *E. senck.* subspecies (Figure 5B).

### Analysis and Interpretation of the Model

Because the chirality gene is maternally expressed, an individual's phenotype may not correspond to its genotype. This means that we needed to follow the proportions of five kinds of snail: Assuming for the moment that the sinistral allele (S) is dominant over the dextral allele (D), sinistral snails may contain SS, SD, or DD genotypes, whereas dextral snails may be SD or DD (Table S4). No dextral snails can have a SS genotype, because all snails with the SS genotype inherit an S allele from their mother, and so are sinistral. The proportions of the five kinds of snail tend to a characteristic equilibrium that can be thought of as analogous to the Hardy-Weinberg proportion (Table S5). Full details of our model are described in the Protocol S1. Here, an overview is given with an emphasis on findings that are directly relevant to *Euhadra*.

**Figure 6:**
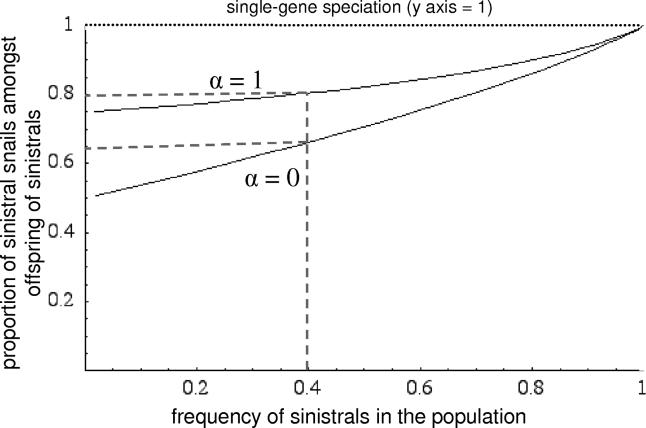
Proportion of Sinistral Snails Amongst Offspring of Sinistrals Compared with Frequency of Sinistrals in the Population The curves are the boundaries defined by extreme values of *α,* the parameter that describes the degree of interchiral mating (lower curve: random mating between chiral morphs, *α* = 0; upper curve: no interchiral mating, *α* = 1). The space between the two curves represents intermediate values of *α*. The intercepts illustrate the predictive nature of the model; e.g., since interchiral mating is not possible in *Euhadra* (*α* = 1), then if a mixed population of *Euhadra* contains 40% sinistrals, the model predicts that 80% of the offspring of sinistral snails should be sinistral. For a species with no barriers to interchiral mating (*α* = 0), the model predicts that 65% of the offspring should be sinistral. Finally, if the mixed population is actually two separate species that might have formed by single-gene speciation, then the prediction is that sinistral snails should always give birth to sinistral offspring (y = 1, indicated by the dotted line; this is the second, unstable equilibrium that is referred to in the main text). Equilibria are calculated from Equation 5 in Protocol S1.

Briefly, the model was used to find the equilibrium frequencies of the chirality gene in each chiral morph, depending upon whether there is random or nonrandom mating (i.e., either interchiral mating with no assortment between chiral morphs, *α* = 0, or complete assortment with no interchiral mating, *α* = 1, respectively). The model is powerful because it predicts the frequency of the sinistral offspring from sinistral morphs as a function of the frequency of sinistral morphs in the population (Figure 6). We expected that the model would be useful in understanding the mitochondrial phylogeny because, by extension, if chirality genes are able to flow freely between chiral morphs, then so will any other gene regardless of whether it is physically linked (on the same chromosome) to the chirality gene or not. Throughout the analysis, we assumed that the allele for sinistral coiling is dominant, as in *Partula,* so the interpretation would have to be modified if dextral is dominant in *Euhadra*.

We were able to account for a variety of other factors as well as those determining whether mating is random or not. They included frequency-dependent selection, in which rare chiral morphs are less likely to find a mate, and selection against hybrid mating, which might occur if snails are less likely to transfer sperm in interchiral mating. We also considered the effects of reproductive character displacement; i.e., a sinistral species would be at a disadvantage if it risked mating with another sympatric sinistral species when hybrid matings between the two species are sterile or when hybrid offspring are less fit. If a new dextral morph arose within a sinistral species, then while it might initially be at a disadvantage (it would have difficulty finding morphs of the same chirality to mate with), the disadvantage could be counterbalanced if the dextral individuals did not waste time mating with the other species. This explanation for the origin of new chiral morphs has been suggested for *Partula* [12], but has not been tested theoretically.

We first investigated the extent of gene flow between different chiral morphs, depending upon their degree of interchiral mating (Section 2 in Protocol S1), using a parameter, *α,* to define the degree of interchiral or assortative mating. Two equilibria are possible in a chirally mixed population. In the first, gene flow between chiral morphs is substantial, sinistral mothers produce a large proportion of dextral offspring (and dextral mothers produce a large proportion of dextrals), and the population rapidly moves to a balanced equilibrium, even if there is absolutely no interchiral mating (*α* = 1). This high degree of gene flow is a direct consequence of the delayed (maternal) expression of chirality. The implication for *Euhadra* is that gene flow between different chiral morphs is inevitable, even if the morphs are themselves unable to mate.

We have illustrated this first equilibrium in Figure 6. In this figure, the axes represent the proportion of sinistrals in the population (x-axis) and the proportion of sinistral offspring from sinistral mothers (y-axis). These two parameters were used to illustrate the model because they represent data that can be gathered in the field, and because they have predictive value. For example, in *Euhadra,* interchiral mating is either not possible (*α* = 1, upper line in Figure 6) or very difficult (for example, when *α* ≈ 0.99). At sites where dextral and sinistral *Euhadra* coexist, we can measure the frequencies of each. If sinistrals are 40% of all snails collected, and different chiral morphs are not able to mate (*α* = 1), then the model predicts that 80% of the offspring of sinistral snails will be sinistral. In contrast, if sinistral and dextral chiral morphs were able to mate freely (*α* = 0; unlikely in *Euhadra*, but possible in other species), then the model predicts that 65% of the offspring of sinistral snails will be sinistral. Finally, if there is no gene flow, then sinistral snails will not give birth to dextral individuals (and vice versa). This is the second equilibrium (the upper dotted line in Figure 6), which corresponds to single-gene speciation, and is discussed below.

The second equilibrium is a special case because it is the only equilibrium in which there is no gene flow between the two morphs, so that they are reproductively isolated. It can only occur if the different morphs are completely unable to mate (*α* = 1). However, this equilibrium is unstable and will return to the first equilibrium (free gene flow between chiral morphs) if any sinistral snails have recessive dextral alleles (or alternatively, dextral snails have dominant sinistral alleles), or if there is a very low frequency of interchiral mating (e.g., *α* ≈ 0.99). The only requirement, therefore, to enable gene flow between different chiral morphs in this second equilibrium is that intrachiral matings occasionally produce offspring of the opposite asymmetry to the mother, or else that interchiral mating occasionally occurs. Intrachiral matings could produce offspring of the opposite asymmetry by either de novo gene mutation or accidents of development, which can happen when embryos develop in extreme environments. At present we do not know the frequency of interchiral mating in species such as *Euhadra*. Although no interchiral matings have been observed, they may be possible (i.e., *α* ≤ 1).

**Figure 7:**
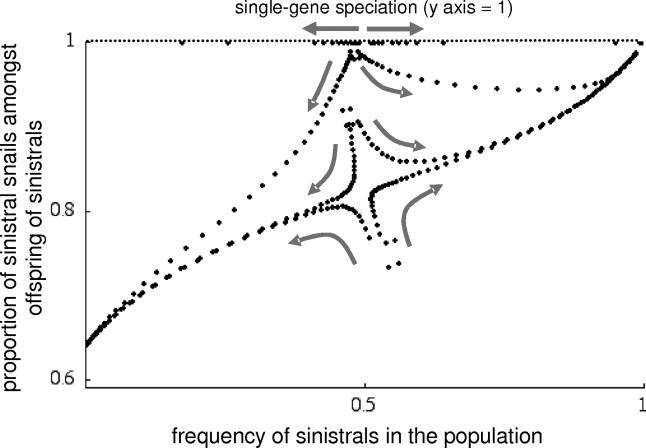
Proportion of Sinistral Snails Amongst Offspring of Sinistrals Compared with Frequency of Sinistrals in the Population, with Reduced Mating Frequency or Fertility for the Rare Chiral Morph We assumed a strong frequency-dependent mating disadvantage against the rare morph, no interchiral mating (*α* = 1) and equal sexual selection on the two sexes. The graph shows eight runs that start close to the border separating two alternative outcomes, which runs approximately vertically down the middle of x-axis of the graph (when the frequency of sinistrals is about 0.5). As there was no interchiral mating, then if the starting condition is two distinct populations (sinistral snails have only sinistral alleles and vice versa), then the two chiral morphs stay separate: the points along the top horizontal line represent this, indicating competition between two reproductively isolated types, or single-gene speciation (y = 1; as in Figure 6, this is also the second, unstable equilibrium that is referred to in the main text). In all other circumstances, the model shows that the population moves to equilibrium, so that there is extensive gene flow between different chiral morphs. For further information, see Results or section 3.i in Protocol S1.

We next considered the effects of a lower frequency or fertility of mating in the rarer chiral morph, giving the more-common morph an advantage (Figure 7; section 3.i in Protocol S1). To simplify the equations, we also assumed that differences between selection on “males” and “females” are not of significant effect, which is reasonable since most snails are hermaphrodites. We imagine that fertility is reduced in interchiral mating because sperm is less likely to be transferred. Perhaps more importantly, an individual of the rare chirality might produce fewer offspring, because it will waste time searching for a suitable mate.

Although the model and its equations became more complicated when the above selective factors were included (section 3.i in Protocol S1), the results were remarkably similar to the first set of circumstances (described above). With random mating (no barriers to interchiral mating, *α* = 0), the population moves close to an equilibrium level of gene flow within two generations, even when selection is close to its maximum value. With complete assortment (no interchiral mating, *α* = 1), the population moves to equilibrium rather more slowly, since gene flow between morphs is somewhat restricted, but eventually reaches a similar position (Figure 6).

We used the same axes as in Figure 6 to illustrate the equilibria that are reached when selection is included (Figure 7). As the description of the outcome is somewhat more complicated, we used the figure to illustrate the extreme condition only, where no interchiral mating is possible (*α* = 1). The graph shows eight trajectories that start close to the boundary separating two alternative outcomes. As there was no interchiral mating, if the initial condition is two separate subpopulations (i.e., sinistral snails contain only sinistral alleles and vice versa), then the two types stay distinct: the population moves along the top horizontal (dotted) line, with competition between two reproductively isolated types. As before, this situation is equivalent to single-gene speciation. However, if there are any chiral morphs that contain the opposite allele, then the population will move towards an apparent equilibrium in which there is extensive gene flow between the morphs (dotted line running from bottom left to top right in Figure 7), irrespective of the starting frequency of chiral phenotypes and genotypes. Once near this quasi-equilibrium, frequency-dependent selection moves the population towards fixation of one or other morph. Under these conditions, the opportunity for single-gene speciation must be very limited, and even more so if interchiral mating is possible (*α* < 1). Moreover, if single-gene speciation does occur, then as before, it is unstable because of the introduction of new chiral alleles or accidental reversals of chirality.

Another consideration was that selection might act against hybrids between different chiral morphs (section 3.ii in Protocol S1), because genetic differences leading to postmating isolation might be held in disequilibrium with the chirality locus itself. While the chirality gene and genes that determine whether hybrids are less fit (or not) are not likely to be physically linked (on the same chromosome), they may be associated because of coincident selection against the physical act of interchiral mating and because the offspring from interchiral matings tend to be less fit. While this seems unlikely because gene flow should be extensive between different chiral morphs (recombination will break down the disequilibrium), selection against hybrids could occur if the different chiral morphs originally evolved in distinct populations and then came together into a single population. In the most extreme circumstance, all heterozygotes would die, interchiral matings would not produce viable offspring, and the system would be stable.

We also considered a more realistic model, in which heterozygotes have their fertility or survival reduced by a fixed proportion (section 3.ii in Protocol S1). As in the former models, except when there is an almost complete lack of interchiral mating (*α* > 0.99), the model shows that there should be high gene flow between morphs, so that heterozygotes are abundant, and selection causes fixation of the more-common allele relatively quickly. When assortment is almost complete (*α* ≈ 1), there is a narrow range of parameters with two alternative ways for the population to reach fixation: either rapidly, with high gene flow between morphs, or much more slowly, with little gene flow. Nonetheless, the outcome in all circumstances (except when all heterozygotes die) is the same: extensive gene flow between chiral morphs.

Up to this point, we assumed a single population, whereas the more-common circumstance may be that most populations are fixed in their chirality, with narrow clines between them, as in *Partula* [12]. The model suggests that regardless of whether mating is random or there is no interchiral mating at all, the position of the cline will move in favour of the dominant allele (section 4.i in Protocol S1) because the dominant allele tends to increase the frequency of its corresponding phenotype following interchiral matings. This conclusion was reached independently by Johnson et al. [25] and Mallet (“dominance drive”) [34]. It is interesting that the shape of the cline will be almost identical regardless of the degree of assortative mating, and in all cases long tails of introgression are expected on either side of the centre of the cline.

A final use of the model was to try to understand the conditions under which new chiral morphs can become established in the first place (section 5 in Protocol S1). If the population were initially entirely sinistral, then positive frequency-dependent selection would act to prevent the establishment of dextral morphs. This is because new chiral morphs would not be able to find a mating partner. However, if a proportion of all matings would otherwise be with another sinistral species, and these matings did not give viable offspring (or the hybrids were less fit), then the mechanical isolation that leads to lower fitness in attempted interspecific interchiral matings would give the new chiral morphs an advantage, because of reproductive character displacement. The model shows that the dextral morph would be at an advantage, even when rare, if at least one-third of matings would otherwise be with another species. Therefore, under conditions where reproductive character displacement is favoured, it is considerably more likely that a new morph will become established. If the new chiral morph is due to a dominant mutation, then it will move forwards in any cline that is created, due to the “dominance drive” mentioned above.

## Discussion

### A Single Evolution of Sinistral Snails

The mitochondrial phylogeny is consistent with a single and relatively ancient origin of sinistral *Euhadra*. After the new sinistral morph became established, five sinistral species evolved, now distributed through central and northern parts of the main Japanese island of Honshu (see Figures 1 and 2). An intriguing question is how the sinistral morph spread and then speciated, despite positive frequency-dependent selection against the rare chiral morph. The focus of most simulations to date has been to understand the relative contributions of genetic drift and gene flow in small populations [23,25–27]. An alternative that could assist the establishment of a new morph is reproductive character displacement. Although this explanation was first suggested by Clarke and Murray in relation to *Partula* [12], it has not been tested theoretically. Reproductive character displacement could be a general explanation for many instances of chiral reversal, including *Euhadra,* the clausilid *Isabellaria,* and *Diplommatina* [35–37]. It could also explain the observation that chirally reversed species are more often found on isolated islands, because they are often inhabited by large clades of closely related species.

In *Partula,* character displacement probably acted so that dextral morphs of otherwise sinistral species such as *P. suturalis* were less likely to hybridize with sympatric, sinistral congeners. In a similar manner, a newly arising sinistral morph in a dextral *Euhadra* species would be less likely to hybridize with other dextral *Euhadra* species. Thus, a common explanation of how a new chiral morph was established in *Partula*, *Diplommatina*, *Euhadra,* and perhaps other species is that initially rare morphs were stabilized against positive frequency-dependent selection by reproductive character displacement. While detailed observations are presently available only for *Partula,* we used the mathematical model to investigate what might be a general mode of divergence. We found that the new morph would be at an advantage even when rare if at least a third of all matings would otherwise be with another species. In these conditions, the new morph would be fixed and begin to spread out from the region in which it originated.

If the new morph went on to establish a population beyond the region of overlap with the other chiral morph, a cline might develop, as presumably happened in *P.* *suturalis* [13]. Moreover, if the allele for the new morph were dominant, then it would tend to move forward due to “dominance drive” [25,34], although movement might be impeded by local barriers. Since the dominance relations in *Euhadra* are unknown, it is difficult to make predictions that are more specific, but in *P. suturalis* the dextral gene is recessive, so dominance drive could have acted against the establishment of a new dextral morph.

An effect of population density could aid the establishment of new morphs. In *Heliconius* butterflies, the interaction between frequency and density is often overlooked as a factor that could assist the establishment of new wing-pattern morphs [29]. At low density, purifying selection against new morphs is strong because predators remain naive, but when the population density is high, selection is weak over a broad range of intermediate frequencies [29]. In *Euhadra,* the parallel situation is that new morphs are less likely to find a mate at low density than at high density. New chiral morphs are therefore more likely to become established in populations at high density, because mates of the same chirality are more easily found.

### Single-Gene Speciation in Sympatry or Mitochondrial DNA Introgression?

The phylogeny did not resolve the relationship between the six major groups within the sinistral clade, as it contains two exclusively dextral groups, three sinistral groups, and one mostly sinistral group that also includes dextral *E. senck. aomoriensis* (see Figures 3 and 4). Recently, Ueshima and Asami found a similar phylogeny using a different set of mitochondrial gene fragments [24]. One interpretation of these phylogenies is that the polyphyly of *E. senck.* *aomoriensis* within the sinistral mitochondrial DNA *E. quaesita* clade is suggestive of recent gene flow between sinistral *E. quaesita* and dextral *E. senck. aomoriensis*. An alternative, as concluded by Ueshima and Asami, is that the polyphyletic *E. senck.* *aomoriensis* lineages are in fact a different (i.e., new) species that has been derived from *E. quaesita* by single-gene speciation [24].

**Figure 8:**
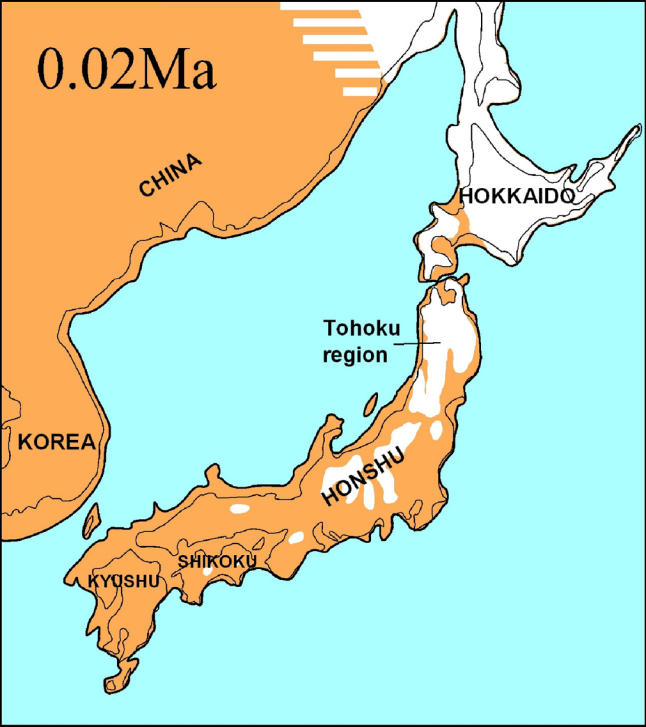
Map of the Periglacial Region in Japan at the Height of the Last Glaciation The last glaciation peaked about 20,000 years ago. Most of northern Tohoku must have been unvegetated (white shading), so *Euhadra* probably colonised from the south in the early Holocene. Brown shading shows regions above sea level, blue is sea, and the black outline indicates present-day Japan. The distribution of the periglacial region in earlier epochs is more uncertain. Data compiled from [56–58].

As in the original evolution of sinistral *Euhadra* that was discussed earlier, initially rare dextral morphs of *E. quaesita* could have been favoured in their spread by reproductive character displacement from another species. The most widespread sinistral species with which *E. quaesita* might have hybridized is *E. decorata,* which is found in the northern part of Tohoku. This explanation is reasonable because *E. quaesita* has a restricted distribution in northern Tohoku, whereas *E. senck. aomoriensis* and *E. decorata* are quite widespread there (see Figure 2). Thus, dextral *E. senck. aomoriensis* may have evolved from sinistral *E. quaesita,* because otherwise *E. quaesita* would tend to hybridize with sympatric, sinistral *E. decorata*. If so, this process could have been quite recent and in situ, because northern Tohoku is considered to have been unvegetated during the last glaciation (Figure 8).

However, it is presently not possible to resolve whether hybridization or single-gene speciation best explains the mitochondrial DNA phylogeny, especially since the dominance of the chirality gene is unknown, and the morphological analysis complicates the interpretation (see Figure 5). We therefore used our model to compare the two competing explanations. We found that when there is even a slight degree of interchiral mating (incomplete assortment; *α* < 1), there is a single feasible equilibrium that is always stable. This equilibrium, which is analogous to a Hardy-Weinberg equilibrium while accounting for the maternal inheritance of the chirality gene, is one of substantial gene flow between the chiral morphs (see Figure 6).

When there is no interchiral mating (complete assortment; *α* = 1), two equilibria become possible. As before, in the first equilibrium there is substantial gene flow between morphs. This is interesting because it means that even when different chiral morphs are unable to mate (as in *Euhadra*), gene flow will still be substantial because of the delayed (maternal) inheritance. This observation was first pointed out by Johnson et al. [25]. In the second equilibrium, there is complete reproductive isolation so it amounts to single-gene speciation, but is only possible when sinistral snails are entirely sinistral homozygotes, and dextral snails are entirely dextral homozygotes. The latter equilibrium is unstable to the introduction of sinistral snails carrying dextral alleles or vice versa, and to a low frequency of interchiral mating. The empirical studies that have been carried out in *Partula* and also *Achatinella* tend to support the predictions from the model of high gene flow between morphs [38–40]. While assortative mating should be much greater in *Euhadra* because interchiral mating is not possible (*α* ≈ 1) [14], the prediction is that gene flow still should be substantial.

Another factor that could reduce gene flow between chiral morphs is selection. First, the more-common morph could be at an advantage because it spends less time searching for its own morph to mate with, and may well have more offspring as a consequence. Second, snails are probably less likely to transfer sperm successfully in interchiral matings. There is direct evidence for reduced fertility in *Partula,* since fewer young are produced by mixed pairs [13]. When we incorporated this mode of selection into the model, with random mating (inter- and intrachiral mating equally likely; *α* = 0) the population moves rapidly to equilibrium gene flow. Even with a complete lack of interchiral mating (*α* = 1), there is still substantial gene flow. The main difference is that the population moves towards equilibrium rather more slowly (see Figure 7).

Although there are no data for *Euhadra,* it is possible that a selection operates against hybrids between chiral morphs because genetic differences leading to postmating isolation are held in association (disequilibrium) with the chiral gene itself, preventing introgression. Evidence from the model suggests that such disequilibrium is unlikely to build up in sympatry. However, if two different morphs met after a period in allopatry, then the population could fall into an equilibrium in which gene flow is reduced, and so maintain the initial association. Even in this circumstance, the model shows that for all but very strong assortment (*α* > 0.99), there still should be high gene flow between morphs, so that heterozygotes would be abundant and selection would cause fixation of the more-common viability allele relatively quickly.

The results from the model have important implications for the interpretation of the mitochondrial DNA phylogeny, and can help to make suggestions for future research. Reproductive character displacement can explain how new chiral morphs arise and become the majority. However, even with reproductive character displacement there will still be a significant flow of the chirality gene (and other genes) between morphs. In these circumstances, speciation by chiral reversal is meaningful only in the context of a complex biogeographical process, which must usually involve other factors. Allopatry, perhaps combined with ecological selection, will be required for genetic differences and reproductive isolation to build up between chiral morphs. As this must have happened in *Euhadra* and several other species, it will be interesting to disentangle the relative contributions of each towards reproductive isolation.

The immediate question is whether sinistral *E. quaesita* reverted back to a dextral species, producing *E. senck. aomoriensis,* or whether there has been gene flow between *E. quaesita* and *E. senck. aomoriensis*. To resolve this issue, a priority should be to investigate the phylogeny of nuclear gene sequences and compare them with the mitochondrial DNA phylogeny. If dextral *E. senck. aomoriensis* arose by single-gene speciation, then most nuclear loci should place these specimens within the *E. quaesita* clade. Alternatively, if the polyphyletic position of dextral *E. senck. aomoriensis* in the mitochondrial DNA phylogeny is due to hybridization and introgression, then the genomes of the two populations should be a mosaic in their extent of differentiation [41–43]. However, even with a complete lack of interchiral mating, introgression and fixation of lineages will occur rapidly, so that introgression may be difficult to distinguish from single-gene speciation.

Another priority should be to establish the dominance relations of the chirality genes in *Euhadra*. This is because a prediction of the model and previous simulations [25,26,34] is that morphs are more likely to be established by single-gene speciation if the mutation involved is dominant. One means to establish dominance would be to attempt breeding experiments in the laboratory. However, there are no known dimorphic species, and interchiral mating has not been observed, so this may not be possible. A solution would be to discover a rare coiling variant, as has been done in *Bradybaena similaris* [24]. The final priority is to finely map the distributions of the different morphs, especially sinistral *E. quaesita,* sinistral *E. decorata,* and dextral *E. senck. aomoriensis,* to test whether the patterns are consistent with reproductive character displacement as an explanation. Individuals from zones of contact could be used in captive breeding experiments to determine the dominance relations, the proportions of each chirality allele, and the extent of gene flow (if any).

### Wider Significance

The interpretation of the model could impact upon the understanding of the evolution of any gene with an indirect genetic effect, especially maternal-effect genes [44]. Maternal-effect genes are important in development because maternally produced molecules (e.g., mRNAs) in the egg affect early developmental processes such as axis formation, and often have later pleiotropic effects. Maternal-effect genes have been implicated in causing infertility [45], with a few also directly implicated in causing hybrid sterility [46], although generally it has been realized that their contribution to postzygotic isolation might be limited [47]. Finally, sex determination in many species involves interactions between maternal and zygote genes, so it has been proposed that conflicting selective pressures between maternally and zygotically expressed sex-determining loci can in turn have a role in shaping the evolution of sex determining systems [48]. However, the detailed discussion of the relevance of our model to these and other issues is beyond the scope of this paper.

### Conclusions

The concept of single-gene speciation in snails has attracted attention because it is an exception to the accepted view that at least two pairs of interacting genes are required for reproductive isolation [21,22,24]. We have shown that reproductive isolation between chiral morphs is possible, but unstable, and will lead almost inevitably to gene flow. While chiral morphs may be a step towards distinct species, complete reproductive isolation must require other factors, such as divergent selection for habitat use or geographic separation of populations. Assuming that the differences in genitalia are not due to dominance or epistasis, chiral morphs with distinct genitalia should be considered “good” species (even if not biological species; [49]), because the morphological distinctness must have been maintained despite gene flow.

## Materials and Methods

### Samples

The 22 species of *Euhadra* (Bradybaenidae) are distributed throughout Japan and the neighbouring Korean island of Jeju [50]. We sampled all five sinistral species for morphological and molecular analysis, in addition to 14 of the 17 dextral species (Figure S1; Tables 1 and S1). The remaining dextral species, *E. sadoensis,* *E. nachicola,* and *E. sigeonis* have restricted distributions. The distributions of four species are shown in Figure 2, using data compiled from [50,51] and the Web site http://www.biodic.go.jp/site_map/site_map.html (in Japanese). We were also able to use an extensive set of 16S rRNA sequences from *E. peliomphala* [52]) and *E. quaesita* [33]. For the outgroup, *Nesiohelix* *bipyramidalis* was used because a prior analysis has suggested that it is suitable [31].

To enable a clear comparison between our results and those of Ueshima and Asami [24], we refer to subspecies where necessary, especially with regard to *E. senck*. Note that Ueshima and Asami refer to *E. senck. aomoriensis* as *“E. aomoriensis”* and *E. murayamai* as *“E. quaesita murayamai”* [24].

### Morphological analysis

Morphological characters were measured in a subset of the species, primarily those from the “sinistral” clade, and then used to make a maximum parsimony tree. In the first analysis of genital shape and structure, the characters were classified as follows: (1) wings on the basal part of dart: 0, absent; 1, small; and 2, large (ordered). (2) Wings on the middle part of dart: 0, absent; 1, small; and 2, large (ordered). (3) Curve of wings on the front part: 0, absent; 1, present. (4) Shape of wings: 0, absent; 1, straight; and 2, curved (ordered). (5) Maximum number of wings: 0, absent; 1, two; and 2, three (ordered). (6) Shape of cross-section of dart: 0, round or oval; 2, triangular; and 1, crescent (ordered). (7) Flatness of the front section of the dart: 0, round; 1, flat; and 2, very flat (ordered). (8) Flatness of the middle section of dart: 0, round; 1, flat; and 2, very flat (ordered). (9) Flatness of the basal section of dart: 0, round; and 1, flat. (10) Shape of the front part of dart-sac: 0, round; 1, oval; and 2, long-oval (ordered). (11) Shape of the mid-dorsal part of dart-sac: 0, curved; and 1, straight. (12) Shape of the dart-sac near atrium: 0, round; and 1, pointed. (13) Length of the middle part relative to the front part of dart-sac: 0, short; and 1, long. (14) Size of the sheath at the basal part of dart-sac: 0, small; and 1, large. (15) Relative size of sub-dart sac: 0, large; 1, medium; and 2, small (ordered). (16) Shape of sub-dart sac: 0, round; and 1, flat. (17) Number of mucus glands: 0, > 10; 1, from 5 to 10; and 2, < 5 (ordered). (18) Region between sub-dart sac and dart sac: 0, not contracted; and 1, contracted.

In the second analysis, that of shell shape and structure, the characters were classified as follows (all sizes in millimetres): (1) size of shell (diameter × height): 0, < 50; 1, 50–60; and 2, > 60 (ordered). (2) Relative height (height/diameter): 0, < 0.5; 1, 0.5–0.6; 2, 0.6–0.7; and 3, > 0.7 (ordered). (3) Relative aperture size ([aperture height + aperture width]/diameter): 0, < 1.0; and 1, > 1.0 (ordered). (4) Uppermost band: 0, missing; and 1, present. (5) Peripheral band: 0, missing; and 1, present. (6) Background colour: 0, pale; and 1, dark. (7) Narrow flare-like colour pattern along the growth line: 0, missing; and 1, present. (8) Wide flare-like colour pattern along spiral line: 0, missing; and 1, present. (9) Growth line: 0, weak; 1, medium; and 2, strong (ordered). (10) Interval of growth line: 0, narrow; and 1, wide. (11) Fine spiral lines: 0, unclear; and 1, clear. (12) Gloss of surface: 0, absent; and 1, present. (13) Small dents irregularly located on the surface: 0, missing; and 1, present. (14) Umbilicus: 0, deeply open; and 1, shallow. (15) Umbilicus: 0, < 1/3 covered by basal lip; and 1, > 1/3 covered by basal lip. (16) Basal lip: 0, attached inside of umbilicus; and 1, attached outside of umbilicus. (17) Shoulder of body whorl: 0, weak; and 1, strong. (18) Peripheral angularity: 0, missing; and 1, present. (19) Inflation of upper part of body whorl: 0, weak; and 1, strong. Shell characters are relatively uniform within *Euhadra* populations, but where necessary, polymorphic characters were coded as an extra character (5).

### DNA extraction and PCR amplification

Genomic DNA was isolated using previously described methods [53]. Primers for PCR amplification of an approximately 900-bp fragment of 16S rRNA are described in [31]. Variation in the mitochondrial 12S rRNA and cytochrome oxidase *c* subunit I genes, and nuclear ITS2 region was also investigated, using primers described in [31,53,54]. All PCR reactions used Takara rTaq (Takara Biomedicals, Tokyo, Japan) and buffers, with annealing temperatures of 50 °C. Cycle sequencing was carried out with both forward and reverse primers, using about 80–100 ng of PCR product in the reaction and the BigDye Terminator v3.0 Cycle Sequencing Ready Reaction Kit (Applied Biosystems, Foster City, California, United States). DNA sequences were electrophoresed on a 310 Genetic Analyzer (Applied Biosystems).

### Phylogenetic analyses

Sequences were aligned using the ClustalX software, and then checked manually. All insertion and deletion sites (indels), as well as several difficult-to-align regions, were removed before phylogenetic analysis. Phylogenetic relationships were analyzed using two methods: neighbour-joining and maximum likelihood, both with PAUP*4.0b8 [55]. Multiple hits were corrected using the general time reversible (GTR) model. The rate matrix, base frequencies, and shape parameter *(α)* of the gamma distribution (based on 16 rate categories) were estimated using likelihood, by iteration from an initial neighbour-joining tree. Parameters estimated from the initial tree were used to make a new neighbour-joining tree. The parameters were then re-estimated, and the process was repeated until there was no further improvement in likelihood. Bootstrap values were then calculated using 1,000 replicates. Maximum likelihood methods used a heuristic procedure with tree-bisection-reconnection, and 100 bootstrap replicates.

### Assumptions

In the snails that have been investigated, asymmetry (or chirality) is under control of one or a few linked loci, where the phenotype of the offspring is controlled by the maternal genotype (maternal inheritance). Dominance is variable, even within genera: dextral is dominant in *Lymnaea peregra, L. stagnalis,* *B. similaris,* and *P. mirabilis* [5,7,8,10,11,24], but sinistral is dominant in *P. suturalis* and *Laciniaria* [9,25]. Reversed asymmetry can sometimes be agenetic, when development is disrupted due to environmental extremes, usually heat or cold shock. We assumed that asymmetry is also maternally inherited in *Euhadra*. In *Partula,* mating is possible between different morphs (although it occurs at reduced frequency) because they are high-spired and mate by shell-mounting [19]. There is no such detailed information for *Euhadra,* but evidence suggests that interchiral mating is difficult and perhaps impossible, because they mate “face-to-face” [14].

### Mathematical model

We developed a mathematical model to understand gene flow between chiral morphs, as well as the route by which new chiral morphs can become established. The methods are described in detail in Protocol S1.

## Supporting Information

Protocol S1 The Mathematical Model (89 KB DOC).

Figure S1 Sample Sites Used in This Study The sample sites used in this study, highlighting in particular the sites where *E. quaesita*, *E. murayamai* and *E. senckenbergiana* were collected (underlined if used for DNA methods). m, *E. murayamai;* q, *E. quaesita;* sa, *E. senck. aomoriensis;* si, *E. senck. ibukicola;* sm, *E. senck. minoensis;* sn, *E. senck. notoensis;* ss *E. senck. senck.*
(662 KB TIF).

Table S1 Species Collected at Each Site (24 KB DOC).

Table S2 Genital Characters See Materials and Methods for definitions.
(24 KB DOC).

Table S3 Shell Characters See Materials and Methods for definitions.
(27 KB DOC).

Table S4 The Five Classes of Snail and Their Frequencies The formulas used in calculating frequencies were *Q* + *P* = 1, and *d* + *s* = 1.
(33 KB DOC).

Table S5 The Contribution of Each of the Four Phenotypic Mating Combinations to the Next Generation Note that *α* describes the strength of assortative mating, *β* describes the mating advantage of dextral over sinistral snails (averaged over the sexes), and *Δ* describes the difference in mating advantage between the reciprocal crosses. Necessarily, *α* < 1−|*Δ*|/2.
(25 KB DOC).

## Abbreviations

D: dextral allele
S: sinistral allele
