# Supplementary material for: Speciation and Gene Flow between Snails of Opposite Chirality
Source: PLoS Biol. 2005 Aug 9;3(9):e282. doi: 10.1371/journal.pbio.0030282 (PMC1182688; doi:10.1371/journal.pbio.0030282)
Supplement: Supplementary file 1 [file pbio.0030282.sd001.doc]

**1. The model**

i) *The state of the population*

Left-right asymmetry (or chirality) in snails is determined by a single genetic locus (the chirality gene), where the phenotype of the offspring is controlled by the maternal genotype (maternal inheritance). There are therefore three genotypes (SS, SD, DD) which determine two chiral morph phenotypes (sinistral SS, SD and dextral DD), potentially giving six kinds of snail. In reality however, there are only five classes of snail: since an SS homozygote must have received an S allele from its mother, and the S allele is dominant in our model, then all such snails must be sinistral.

Under these circumstances, the frequency of the different classes can be represented by four parameters, *P*, *s*, *su*, *w* (Table S4):

- *P*, *Q* are the frequencies of the sinistral and dextral alleles, respectively (*Q*+*P* = 1).
- *s*, *d* are the frequencies of the sinistral and dextral chiral morphs, respectively (*d*+*s* = 1).
- *su* is the frequency of SS homozygotes with a sinistral phenotype.
- *w* is the difference in frequency of the sinistral allele between the sinistral and dextral phenotypes; it is a measure of the heritability of chirality.

The choice of parameters that we use is somewhat arbitrary. *P*, *s*, and *w* have a clear interpretation, whilst *su* was chosen because it leads to reasonably simple equations. Note that *u* (i.e. *su*/*s*) is the frequency of SS homozygotes within the class of sinistral chiral morphs, but it is simpler to treat *su* as a single parameter. Field data consist of observations of the proportion of sinistral snails (*s*), and of the proportion of sinistrals in the offspring of sinistral snails (*x*; Fig. 6). This latter parameter is related to the others by:

x =	(1)

# ii) *Selection and assortative mating*

To calculate the change in composition of the population from one generation to the next, it is necessary to specify the contribution of each of the 25 possible matings to the next generation. In particular, two processes need to be modelled: assortative mating between chiral morphs (i.e. the degree of interchiral mating) and frequency-dependent selection favouring the commoner chiral morph. Such selection might arise for three reasons: a) genes which had diverged between the races so as to produce post-mating isolation might be held in disequilibrium with the chirality gene, so that SD heterozygotes produce fewer offspring; b) an individual of the rarer chirality might waste more time searching for its own type of mate, and therefore produce fewer offspring. c) sinistral x dextral courtships might fail to transfer sperm as effectively. The first possibility seems unlikely because, as shown later, there is substantial gene flow between sinistral and dextral chiral morphs even when they are unable to mate. Unless the relevant genes are very tightly linked to the chirality locus, disequilibrium is likely to be weak, a point will be discussed in more detail later. The second and third possibilities can be represented quite generally by three parameters:

- *α* (degree of assortment)
- *βM* (relative mating advantage of dextrals acting as males)
- *βF* (relative mating advantage of dextrals acting as females)

It is convenient to define *β* = *βM* + *βF*, which is the net mating advantage of dextrals, and *Δ* = *βM*-*βF*, which is the difference in sexual selection between males and females. The contribution of each of the four phenotypic mating types is given by Table S5, with 0 ≤ *α* ≤ 1-|*Δ*|/2, *β* ≤ 2, which ensures that all contributions remain positive. These parameters may depend on the frequency of sinistrals, *s*. By symmetry, *α*(*s*) = *α*(*d*), and *β*(*s*) = -*β*(*d*) (provided that the presence of other species of snail does not introduce any unevenness: see later).

The theoretical analysis will deal mainly with the parameters *α*, *β*, *Δ*, since this representation leads to relatively simple equations. However, to relate the results to natural populations, some particular form for the frequency-dependence must be assumed. One possibility is that female fertility is simply proportional to the number of sperm transferred. Assuming large numbers of matings, and random use of stored sperm:

α = β = Δ = 0,	(2a)

where *γ* is the fixed proportion of interchiral matings that fail. Alternatively, if female fertility does not depend on the number of successful matings, the selection on snails acting as males will be stronger than that acting on snails as females, so that *Δ* > 0:

α =	$\begin{matrix} \underline{\gamma(1-\gamma(1-2\text{sd}))} \\ 4(1-\gamma+\gamma^{2}\text{sd)} \end{matrix}$ β =	$\begin{matrix} \underline{\gamma(1-\gamma)(\text{d}-\text{s})} \\ 2(1-2sd)(1-\gamma+\gamma^{2}sd) \end{matrix}$ (2b)

In reality, one would expect parameters lying somewhere between these two extreme cases. In both these cases, the net selection pressure in favour of dextrals, *β*, tends to zero when the population is almost monomorphic. This may make it easier for a rare type to establish itself.

iii) *Evolution of the population*

The recursions for the change in the population from one generation to the next can be found by summing over the contributions from each of the 25 possible matings. Some straightforward algebra leads to:

WP^*^ = P	+	(3a)

Ws^*^ = 2P - su	+	+	(3b)

Wsu^*^ = P^2^ +	+ P(Pδ - 2w)	(3c)

Wφ^*^ = P(2P - su) +	+	+	(3d)

w^*^ =	(3e)

where *δ* = 1-2*s* and *W*= 1+(*β*/2)*δ*.

## **2. Neutral dynamics**

The behaviour of the system is best understood by considering first the relatively simple case where there is assortative mating (*α* > 0), but no fitness differences between chiral morphs (*β*, *Δ* = 0). The allele frequency then remains constant, whilst the remaining three variables evolve towards an equilibrium which is analogous to the Hardy-Weinberg equilibrium.

i) *Equilibria*

An equilibrium solution of Eq. 1 for *β*, *Δ* = 0 is given by:

su = 2P - s	(4a)

αw^2^ = sd(d - Q^2^)	(4b)

Pd +2(1-Q2 - s) = w	(4c)

This does not have a useful explicit solution for arbitrary *α*, but does simplify for the extreme cases, *α* = 0 and 1:

A	or	B

(α = 0)	su = P^2^	(α = 1)	su = 2P-s	su = P

s = 1 - Q^2^	s =	s = P	(5)

w =	w =	w = PQ

With incomplete assortment (*α* < 1) there is a single feasible equilibrium, which is always stable (see Appendix). This equilibrium is plotted in Figure 6 for the extreme cases of *α* = 0 and *α* = 1. It is described using the variables (*s*, *x*), since these correspond to the observed data that can be gathered easily (recall that *x* is the frequency of sinistrals amongst offspring of sinistral snails; Eq. 1). When there is complete assortment (*α* = 1), two equilibria become possible (A, B in Eq. 5). In the first (A), there is substantial gene flow between chiral morphs (Figure 6), whilst in the second (B), there is complete reproductive isolation. In the Appendix, we show that complete reproductive isolation (B) is unstable. This result is rather surprising, because it implies that even when there is a complete assortative mating between chiral morphs, there will still be substantial gene flow between these two types. Some sinistral snails will be homozygous for the dextral allele, and some dextral snails will be heterozygous, so that sinistral by sinistral matings will occasionally give dextral offspring, and conversely, some dextral by dextral matings will give sinistral offspring. There is a more obvious equilibrium (B), in which sinistral snails are entirely composed of homozygotes for the sinistral allele, and dextral snails are entirely composed of dextral homozygotes. However, it only exists when phenotypic assortative mating is complete, and, even then, the equilibrium is unstable to the introduction of dextral alleles into sinistral chiral morphs, or *vice versa*.

Since there is considerable gene flow between sinistral and dextral morphs even with complete assortment, the range of possible values of *x* (the proportion of sinistral offspring from sinistral mothers) which can be observed is narrow (at most, 0.5 < *x* < 0.75 for small *s*; Figure 6). This variable therefore gives little information about the degree of assortative mating in the population, essentially because phenotypic assortment has rather little effect on the genetic structure of the population.

## **3. Effect of selection**

When the differences in fitness between the chiral morphs are slight, the population will lie close to the neutral equilibrium trajectories (Figure 6). Selection will simply move the population along the trajectories. This allows a great simplification since it is then only necessary to consider the change of a single variable (*P*); the remaining variables are constrained to follow the neutral trajectory. Numerical solution of the complete set of equations (Eqs. 3) confirms that the above approximation is reasonable.

i) *Incompatibility between chiral morphs*

We concentrate on the effects of reduced fertility of matings between the chiral morphs, and assume that the commoner morph has an advantage of magnitude *b*, defined by *β* = *b*(*d* - *s*). Differences between selection on males and females are likely to have very little effect, so we let *Δ* = 0. When there is no assortative mating (*α* = 0), the population moves close to the trajectory within two generations, and the trajectory is not appreciably distorted even where selection is close to its maximum value (*b* = 2). With complete assortment (*α* = 1), the population moves onto the trajectory rather more slowly, since gene flow between morphs is somewhat restricted (Figure 7). However, the position of the quasi-equilibrium trajectory is similar to that in the neutral case; the variable *x* is slightly reduced when sinistrals are rare, further restricting the influence of assortment on the composition of the population (compare with Figure 6).

ii) *Selection against heterozygotes*

It is possible that there could be selection against hybrids between different chirality genotypes, since genetic differences leading to post-mating isolation might be held in disequilibrium with the chirality locus itself. The previous analysis made this seem unlikely, since it suggested that even with almost no interchiral mating, the population would still lie in an equilibrium where there is considerable gene flow between the chiral morphs. Gene flow and recombination break down any disequilibrium with genes causing hybrid inviability. However, it was also shown that there is another equilibrium in which gene flow is greatly reduced, and so it is possible that if, initially, sufficient hybrid inviability was associated with the chirality locus (as for example if the two races met after a period of allopatric divergence), the population would fall into this upper equilibrium (Eqs. 5, B), and so maintain the initial disequilibrium. Consideration of the extreme case in which all heterozygotes die makes this possibility plausible: any interchiral mating would lead to inviable offspring, and so the system would be stable.

A fully realistic model of the effect of hybrid inviability due to linked loci would be prohibitively complicated. A realistic approximation might be that heterozygotes at the chirality locus have fertility reduced by a factor (1-*ν*), since they would tend to be more heterozygous for linked co-adapted loci, and therefore produce more inviable recombinant offspring. We have examined a still simpler model (See Appendix, Eqs. A4), where the heterozygotes themselves have their viability reduced by a factor (1-*ν*). This gives very similar results for small *ν*.

Results are summarised in the Appendix. For all but very strong assortment (*α* < 0.99), there is high gene flow between morphs, so that heterozygotes are abundant, and selection causes fixation of the commoner allele relatively quickly. When assortment is almost complete, there is a narrow range of parameters in which there are two alternative ways for the population to reach fixation: relatively rapidly, with high gene flow between morphs, or much more slowly, with little gene flow. It is interesting that a system which can be specified so simply gives rise to such complex behaviour. However the range of parameters in which two routes to fixation can coexist is extremely limited. Assortment must be almost complete (*α* ~ 0.999), selection against heterozygotes moderate (0 < *ν* < 0.15), and, furthermore, selection against phenotypic interchiral mating must be low, since it will tend to make the upper equilibrium less stable (*β* << 1). Even if these conditions were met, it seems likely that random drift would obscure the distinction between the two equilibria.

## **4. Clines between dextral and sinistral morphs**

The above treatment dealt with the evolution of chiral morphs within a single population. Selection against the rarer morph will lead to fixation of one or other morph in the population. However, though most snail populations are monomorphic, in some species (e.g. *Partula*) narrow hybrid zones are found between areas fixed for dextral and sinistral morphs. In order to understand how new chiral morphs might spread out after being established in a population, we need to understand the behaviour of these zones.

i) *Selection against interchiral mating*

For weak selection pressures (*β* << 1), we can assume that the population lies close to the trajectory defined by Eq. 2. When *β* is small we can neglect the slight reduction in the proportion of heterozygotes, caused by the Wahlund effect. Since we can find no explicit solution to this equation when 0 < *α* < 1, we consider the two extreme cases of *α* = 0 or 1. As assortative mating will tend to increase the difference in allele frequency between the two morphs, the effect will be to strengthen selection and so sharpen the hybrid zone. The results given here will therefore give upper and lower limits to the cline width.

Modelling gene flow by diffusion at a rate *σ*^2^ (the variance in parent-offspring distances), we obtain

= - f(P)	(6)

where f(P) =

If *β* increases through zero as the frequency of dextral snails increases, so that two stable states are possible within a single population, then a hybrid zone can be set up between a region where *P* = 0 and a region where *P* = 1. Two features of this solution are of interest: the width (defined as the inverse of the maximum gradient), and movement. In a uniform environment, the hybrid zone will move in favour of the dextrals if > 0, and in the opposite direction otherwise. Now, if the environment is symmetrical, we would expect that *β*(*s*) = −*β*(*d*). Using this condition, we find that both when *α* = 0 or *α* = 1, the dominant sinistral allele will be favoured, and will move forward. A similar result reached by different means has been reported previously [1,2].

The width of the cline may be determined by integrating Eq. 6. If we take *β* = *b*(*d* - *s*), (*b* << 1), then, when *α* = 0, the width of the phenotypic cline is 6.07, whereas when *α* = 1, the width is almost identical: 6.19It would be more realistic to use the frequency-dependent form for *β*, in Eq. 2, which was derived by assuming that a fixed proportion (*γ*) of all interchiral matings fail. However, here the degree of assortment necessarily varies with the strength of selection, making the equation very complicated. Since the degree of assortment seems to make remarkably little difference to the cline width, we can get an approximate solution by taking *α* = 0; the width is now 6.15 . Although the maximum gradient here is similar to simpler selection schemes, the shape of the cline is not. Since the selection coefficient is proportional to *sd* (Eq. 2a), there will be long, slowly decaying tails of introgression on either side of the cline with this model.

ii) *Selection against heterozygotes*

Provided that assortment is not too high, this case will give rather similar results to that considered above. When selection is weak, the population will remain near the neutral trajectory, and a hybrid zone will form with width of approximately ; with random mating we have simple heterozygote unfitness, which gives a cline width of . With moderately strong assortment this will remain true for sufficiently small ν, but when heterozygote unfitness increases above some value (*ν* > 0.15 for *α* = 0.90, say), gene flow will be much reduced, heterozygotes will almost disappear, and so the cline will become very much wider. When assortment is almost complete (*α* > 0.999), there is the possibility of two distinct equilibria for the same value of *ν* (see Appendix); the hybrid zone could then either be sharp or broad, depending on initial conditions (If two sections of zone with different stable equilibrium shapes met, they would remain separated by a distinct, stable transition. This possibility is, however, hardly likely to be of practical significance). In nature, selection against interchiral mating is likely to be the dominant factor maintaining the hybrid zone. Other incompatibilities may reduce the level of gene flow, but are unlikely to affect the cline width very greatly.

## **5. Establishment of the dextral morph**

How might the dextral morph have established itself? If the population were initially entirely sinistral, then any rare dextral morph would have been selected against because it would almost always have to mate with shells of opposite coil. However, if a proportion *μ* of all matings were with another dextral species, and if these matings gave no viable offspring, then the mechanical isolation which leads to lower fitness in interchiral matings would give dextrals an advantage. The selection coefficient is now given by:

β = 4γsd	(7)

Thus, the dextral morph will be at an advantage even when rare if at least 1/3 of all matings would otherwise be with a sinistral species. (This is because when *d* = 0, Eq. 7 is positive if *μ* > 1/3). However, the selection pressure will still be weak, since it is proportional to *sd*. The dextral morph will be fixed rapidly in the populations and begin to spread out from the region in which the sinistral species is sufficiently abundant to allow its initial establishment. Once it is beyond this region, a hybrid zone will be established. Subsequent movement of the cline will depend on dominance drive (see previous section; [1]).

**Appendix**

**1. Stability of equilibria**

Since there are two equilibria when *α* = 1, it is important to know which (if any) of these is stable. Let *s* = *s­o*+*σ*, *su* = *suo*+*ε*, *w* = *wo*-*θ*, where *so*, *suo*, *wo* are the equilibrium values, given by Eqs. 5. The response to a small perturbation (*σ*, *ε*, *θ*) away from the mixed equilibrium A (Eqs. 5) is:

$\begin{matrix} \left(\begin{matrix} \sigma\\ \varepsilon\\ \theta\end{matrix}\right)^{*}=\left(\begin{matrix} 0 & -1 & 0 \\ -\frac{\delta_{0}}{4} & 0 & -1 \\ \frac{1}{4}\left(2\frac{\delta_{0}}{s_{0}}P+3s_{0}-2\right) & \frac{1}{2}\left(P-\frac{\delta_{0}}{2}\right) & \frac{P}{s_{0}}-1 \end{matrix}\right)\left(\begin{matrix} \sigma\\ \varepsilon\\ \theta\end{matrix}\right) \\ \\ \end{matrix}$ (A1)

Numerical calculation shows that the eigenvalues of this matrix always have magnitudes less than 1 (|*λ*|max = 0.9816 at *so* = 0), and so this equilibrium is always stable. The response to a small perturbation (σ, ε, θ) away from equilibrium B (complete isolation, Eqs. 5) is:

$\left(\begin{matrix} \sigma\\ \varepsilon\\ \theta\end{matrix}\right)^{*}=\left(\begin{matrix} u & -1 & u \\ P-Q & 0 & -2 \\ \frac{1}{2}\left(1-3P\right) & \frac{1}{2}\left(Q-P\right) & \frac{3}{2} \end{matrix}\right)\left(\begin{matrix} \sigma\\ \varepsilon\\ \theta\end{matrix}\right)$ (A2)

This matrix has eigenvalues 1, 1, -1/2: since the largest eigenvalue is 1, the stability of the equilibrium depends on second order terms. To examine these, we represent the population in terms of eigenvectors of the above matrix. Let *a* = *Qσ* + *ε* + *θ*, *g* = *σ*(1-4*P*)+*ε* +4*θ*. Then, to leading order:

a^*^ = -	(A3a)

g^*^ = g + $\left(\begin{matrix} \underline{\sigma} \\ 3\text{P} \end{matrix}\right)$(4a - g - 3σ)(P - Q)	(A3b)

σ^*^ = σ +	(A3c)

The component a will rapidly tend to zero, and can be ignored. The component *σ* will change in proportion to component *g*; since *g* is only affected by second order terms (~ *σ*(3*σ*+ *γ*)), a slight fluctuation in *g* will cause a prolonged and hence large change in *σ*. This will then act on *g* to produce a further change proportional to -*σ*^2^(*P*-*Q*)/*P*. Numerical solution of both the perturbation equations (Eqs. A3) and the full Eqs. 3 confirm that equilibrium B (Eqs. 5) is never stable.

Although numerical results (e.g. see Figure 6) suggest that it is reasonable to regard the evolution of the population as consisting of a rapid movement towards a quasi-equilibrium state, at a rate determined by the degree of assortative mating, followed by movement under the influence of selection, there is one potential problem. It is conceivable that when *α* = 1, equilibrium B, which corresponds to complete reproductive isolation between sinistrals and dextrals, might be stabilised by the introduction of selection; the population could then fall into two possible quasi-equilibrium states. However, consideration of the stability matrix shows that this equilibrium will in fact be made unstable by most structural perturbations. Since the matrix has eigenvalues (1, 1, -1/2), its characteristic equation has the form (*λ* – 1)^2^(*λ*+1/2) = 0. A change in the matrix proportional to some weak selection pressure will alter this equation to (*λ* – 1)^2^(*λ*+1/2) = *ε γ*(*λ*). If *γ*(1) is positive, then the leading eigenvalues will be perturbed to *λ* = 1±. Since one of these must have magnitude greater than 1, the equilibrium is now unstable. On the other hand, if *γ*(1) is negative, the leading eigenvalues will be perturbed to *λ* = (1+(*ε*/3)(*γ*'(1)-2*γ*(1)/3))±. These both have magnitude |*λ*| = 1+(2*ε*/9)(3*γ*'(1)-5*γ*(1)). So, if *γ'*(1) < (5*γ*(1)/3), the new equilibrium will be stable. The perturbation due to infertility of interchiral mating (*β*) gives *γ*(1) > 0 for all *P*, and so equilibrium B becomes less stable. This is confirmed by numerical results.

**2. Selection against heterozygotes**

After assortative mating, the offspring will be a frequencies given by Eq. 3 (denoted by *). The adult proportions after selection against heterozygotes will then be:

WP^**^ = P^*^ - ν(P^*^ - su^*^)	(A4a)

Ws^**^ = s* - 2ν(s* P*+w^*^-su^*^)	(A4b)

Wsu^**^ = su^*^	(A4c)

W= (2w^*^-δ* P^*^) - ν(2w^*^ - δ^*^P^*^ - su^*^)	(A4d)

where *W*= 1-2*ν*(*P*^*^-*su*^*^)

We first discuss the stability of the two equilibrium trajectories found in the neutral case after the introduction of some small heterozygote disadvantage. The lower equilibrium was stable for all allele frequencies, and all values of *α*, and so slight selection against heterozygotes should not make it unstable. However, when sinistrals are rare, the magnitude of one of the eigenvalues approaches very close to 1 (0.980 for *s* = 0), and so we might expect this equilibrium to become unstable for some small, though finite, value of *ν*, when sinistrals are rare. The upper equilibrium (which only exists for complete assortment in the neutral case) had a leading eigenvalue of 1, and so we might expect that very slight heterozygote advantage would make it stable. Furthermore we would expect that a slight heterozygote disadvantage ν might allow a stable quasi-equilibrium trajectory to exist for incomplete assortment (*α* > *αc* ~ 1-*ν*); the upper equilibrium could then be structurally as well as dynamically stable.

Analysis of the recursions (Eqs. 3, A4) shows that this is indeed the case for weak heterozygote disadvantage (*ν* << 1). A hybrid population can fall into two alternative states, which involve quite different levels of gene flow, and which could be distinguished in nature by the correlation between genotype and phenotype, and by the width of the hybrid zone between sinistrals and dextrals. Our analysis shows that with complete assortment and slight heterozygote disadvantage, the mixed equilibrium (Eqs. 5 A) is stable when dextrals are more common, but becomes unstable when dextrals are rare. The population then moves to complete isolation (Eqs. 5 B), and since there are then no heterozygotes, it remains dimorphic. Thus, a population may reach one of three states: dextrals fixed, sinistrals fixed, or a stable polymorphism in which the morphs are completely isolated from each other.

We have examined the qualitative behaviour when there is strong heterozygote disadvantage (*ν* ~ 1) and strong but incomplete assortment (*α* ~ 1). Since interchiral mating continually generates heterozygotes, selection pushes the population towards fixation for either sinistral or dextral morphs. For moderate assortment (*α* = 0.75 say), there is a single unstable equilibrium; with moderate selection (*ϖ* = 0.5), there is high gene flow between morphs (*x* ~ 0.8), whilst for stronger selection against heterozygotes, gene flow is much reduced (*x* ~ 1). As assortment becomes stronger, lower values of heterozygote unfitness suffice to reduce gene flow: when *α* = 0.99, the two morphs will be effectively isolated when *ν* > 0.15. The behaviour of the system changes qualitatively when assortment is virtually complete; when *α* = 0.999, there is a single unstable equilibrium corresponding to high gene flow (*x* = 0.8 – 0.9) when *ν* < 0.127, and a single equilibrium corresponding to low gene flow (*x* ~ 0.98 – 1) when *ν* > 0.155. However, there is now a narrow range of (0.127 < *ν* < 0.155) for which three unstable equilibria are possible. When assortment is complete, this region expands to 0 < *ν* < 0.152; in this extreme case, the population can either be in a neutral polymorphism with complete genetic isolation, no heterozygotes, and hence no selection, or alternatively, it can fall into a quasi-equilibrium with high gene flow, a substantial proportion of heterozygotes, and hence rapid selection for fixation of one or other morph.

**References**

1. Mallet J (1986) Hybrid zones of *Heliconius* butterflies in Panama and the stability and movement of warning color clines. Heredity 56: 191-202.

2. Johnson MS, Clarke B, Murray J (1990) The coil polymorphism in *Partula suturalis* does not favor sympatric speciation. Evolution 44: 459-464.
