# Supplementary material for: Speciation and Gene Flow between Snails of Opposite Chirality
Source: PLoS Biol. 2005 Aug 9;3(9):e282. doi: 10.1371/journal.pbio.0030282 (PMC1182688; doi:10.1371/journal.pbio.0030282)
Supplement: Supplementary file 6 [file pbio.0030282.st005.doc]

|  |  | ♂ | |
| --- | --- | --- | --- |
|  |  | S | D |
| ♀ | S | s^2^(1 - \f(b,2)) + asd | sd(1 + \f(D,2)) - asd |
|  | D | sd(1 - \f(D,2)) - asd | d^2^(1 + \f(b,2)) + asd |
